# 2-Hy­droxy-5-[(*E*)-(1*H*-indol-3-yl­methyl­idene)aza­nium­yl]benzoate

**DOI:** 10.1107/S1600536810048579

**Published:** 2010-11-27

**Authors:** M. Nawaz Tahir, Hazoor Ahmad Shad

**Affiliations:** a Department of Physics, University of Sargodha, Sargodha, Pakistan; b Department of Chemistry, Govt. M. D. College, Toba Tek Singh, Punjab, Pakistan

## Abstract

The zwitterionic title compound, C_16_H_12_N_2_O_3_, was obtained as a result of the condensation of 5-amino­salicylic acid and 1*H*-indole-3-carbaldehyde. The whole mol­ecule is roughly planar: the 4-hy­droxy­anilinic group and the 1*H*-indole-3-carbaldehyde moieties are only slightly twisted, making a dihedral angle of 7.77 (11)°, whereas, the carboxyl­ate group makes a dihedral angle of 3.34 (45)° with the parent 4-hy­droxy­anilinic group. *S*(6) ring motifs are formed due to intra­molecular O—H⋯O hydrogen bonding. In the crystal, inter­molecular N—H⋯O and C—H⋯O hydrogen bonds build up pseudo-rings with *R*_1_^2^(4), *R*_2_^1^(7) and *R*_2_^2^(14) ring motifs. These pseudo-dimers are further linked by N—H⋯O hydrogen bonds into a chain extending along [101]. C—H⋯π inter­actions also occur, along with offset π–π inter­actions between the anilinic phenyl and the heterocyclic five-membered rings with a centroid–centroid distance of 3.5716 (19) Å.

## Related literature

For background to our ongoing work on the synthesis and ligand properties of Schiff bases derived from 2-hydroxy-5-aminobenzoic acid, see: Tahir *et al.* (2010**a* ▶,b* ▶). For graph-set notation, see: Bernstein *et al.* (1995 ▶); Etter (1990 ▶).
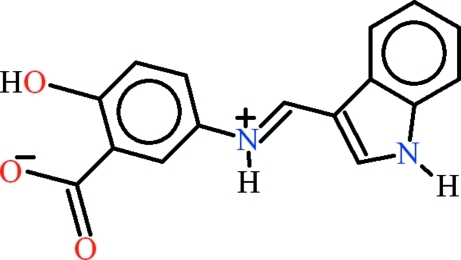


## Experimental

### 

#### Crystal data

C_16_H_12_N_2_O_3_
*M* *_r_* = 280.28
Monoclinic, 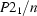

*a* = 7.3463 (6) Å
*b* = 15.5496 (12) Å
*c* = 11.5310 (8) Å
β = 104.619 (4)°
*V* = 1274.57 (17) Å^3^
*Z* = 4
Mo *K*α radiation
μ = 0.10 mm^−1^
*T* = 296 K
0.24 × 0.14 × 0.12 mm

#### Data collection

Bruker Kappa APEXII CCD diffractometer
Absorption correction: multi-scan (*SADABS*; Bruker, 2005 ▶) *T*_min_ = 0.980, *T*_max_ = 0.988
9888 measured reflections
2313 independent reflections
1169 reflections with *I* > 2σ(*I*)
*R*_int_ = 0.080

#### Refinement

*R*[*F* ^2^ > 2σ(*F* ^2^)] = 0.057
*wR*(*F* ^2^) = 0.152
*S* = 1.00
2313 reflections
191 parameters
H-atom parameters constrained
Δρ_max_ = 0.19 e Å^−3^
Δρ_min_ = −0.22 e Å^−3^

### 

Data collection: *APEX2* (Bruker, 2009 ▶); cell refinement: *SAINT* (Bruker, 2009 ▶); data reduction: *SAINT*; program(s) used to solve structure: *SHELXS97* (Sheldrick, 2008 ▶); program(s) used to refine structure: *SHELXL97* (Sheldrick, 2008 ▶); molecular graphics: *ORTEP-3 for Windows* (Farrugia, 1997 ▶) and *PLATON* (Spek, 2009 ▶); software used to prepare material for publication: *WinGX* (Farrugia, 1999 ▶) and *PLATON*.

## Supplementary Material

Crystal structure: contains datablocks global, I. DOI: 10.1107/S1600536810048579/dn2627sup1.cif

Structure factors: contains datablocks I. DOI: 10.1107/S1600536810048579/dn2627Isup2.hkl

Additional supplementary materials:  crystallographic information; 3D view; checkCIF report

## Figures and Tables

**Table 1:** Hydrogen-bond geometry (Å, °) *Cg*3 is the centroid of the C10–C15 ring.

*D*—H⋯*A*	*D*—H	H⋯*A*	*D*⋯*A*	*D*—H⋯*A*
N1—H1⋯O1^i^	0.86	1.90	2.735 (3)	162
N2—H2⋯O3^ii^	0.86	1.95	2.805 (3)	171
O3—H3⋯O2	0.82	1.70	2.448 (3)	150
C16—H16⋯O1^i^	0.93	2.48	3.279 (4)	144
C16—H16⋯O2^i^	0.93	2.55	3.439 (4)	160
C5—H5⋯*Cg*3^iii^	0.93	2.92	3.643 (4)	136

Symmetry codes: (i) 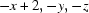; (ii) 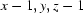; (iii) 
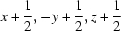
.

**Fig. 1.**
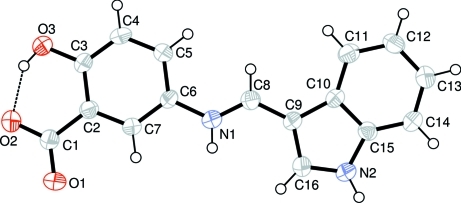
View of the title compound with the atom numbering scheme. The thermal ellipsoids are drawn at the 50% probability level. H-atoms are shown by small circles of arbitrary radii. The dotted line represent the intramolecular H-bonding.

**Fig. 2.**
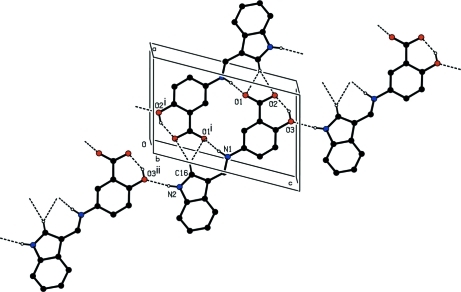
Partial packing view showing the formation of dimers through different ring motifs and infinite one dimensional polymeric chains. [Symmetry codes: (i) -x+2, -y, -z; (ii) x-1, y, z-1]

## supplementary crystallographic information

### Comment

As a part of our on going project related to synthesize various Schiff bases of 2-hydroxy-5-aminobenzoic acid and then their metal complexation (Tahir *et al.*, 2010**a*,b*), we report here the title compound (I).

The title compound (I) is Zwitterion similar to 2-hydroxy-5-{[(*E*)-4-methoxybenzylidene]azaniumyl}benzoate (Tahir *et al.*, 2010*b*). In (I), the group A (C2—C7/N1/O3) of 5-aminosalicylic acid moiety and the 1*H*-indole-3-carbaldehyde moiety B (C8—C15/N2) are almost planar with r. m. s. deviations of 0.0258 and 0.0175 Å, respectively (Fig. 1). The dihedral angle between A/B is 7.77 (11)°. The carboxylate group C (O1/C1/O2) is oriented at a dihedral angle of 3.34 (45)° with the parent group A. The observed values of C═N and C═O are 1.298 (5) and 1.254 (4) Å, respectively compared to 1.291 (2) and 1.242 (2) Å observed in related compound (Tahir *et al.*, 2010*b*).

In the title compound S(6) ring motif (Etter, 1990; Bernstein *et al.*, 1995), is formed due to intramolecular H-bonding of O—H···O type (Table 1, Fig. 1). The N—H···O type of H-bondings build up a pseudo dimer with *R*_2_^2^(14) ring motifs (Table 1, Fig. 2). The C—H···O and N—H···O types of H-bondings (Table 2) complete *R*_1_^2^(4) and *R*_2_^1^(7) ring motifs (Table 1, Fig. 2). The pseudo dimers are interlinked due to H-bonding of N—H···O type (Table 1, Fig. 2) resulting in dimensional polymeric chains extending along the crystallographic [1 0 1] axis. A C—H···π (Table 1) and offset π–π interaction between the benzene ring (C2—C7) of anilinic and heterocyclic five membered (N2/C15/C10/C9/C16) groups at a distance of 3.5716 (19) Å, with plane to plane distance of 3.25 and 3.41 Å and mean offset angle of 20.9°, play important role in stabilizing the molecules.

### Experimental

Equimolar quantities of 1*H*-indole-3-carbaldehyde and and 5-amino-2-hydroxybenzoic acid were refluxed in methanol along with few drops of acetic acid as catalyst for 30 min resulting in orange yellow solution. The solution was kept at room temperature which affoarded orange needles after five days.

### Refinement

The H-atoms were positioned geometrically (O–H = 0.82, N–H = 0.86, C–H = 0.93 Å) and were included in the refinement in the riding model approximation, with *U*_iso_(H) = 1.2*U*_eq_(C, N, O).

### Crystal data

**Table:**

C_16_H_12_N_2_O_3_	*F*(000) = 584
*M**_r_* = 280.28	*D*_x_ = 1.461 Mg m^−^^3^
Monoclinic, *P*2_1_/*n*	Mo *K*α radiation, λ = 0.71073 Å
Hall symbol: -P 2yn	Cell parameters from 1169 reflections
*a* = 7.3463 (6) Å	θ = 2.3–25.3°
*b* = 15.5496 (12) Å	µ = 0.10 mm^−^^1^
*c* = 11.5310 (8) Å	*T* = 296 K
β = 104.619 (4)°	Needle, orange
*V* = 1274.57 (17) Å^3^	0.24 × 0.14 × 0.12 mm
*Z* = 4	

### Data collection

**Table:**

Bruker Kappa APEXII CCD diffractometer	2313 independent reflections
Radiation source: fine-focus sealed tube	1169 reflections with *I* > 2σ(*I*)
graphite	*R*_int_ = 0.080
Detector resolution: 8.20 pixels mm^-1^	θ_max_ = 25.3°, θ_min_ = 2.3°
ω scans	*h* = −8→8
Absorption correction: multi-scan (*SADABS*; Bruker, 2005)	*k* = −18→18
*T*_min_ = 0.980, *T*_max_ = 0.988	*l* = −13→13
9888 measured reflections	

### Refinement

**Table:**

Refinement on *F*^2^	Primary atom site location: structure-invariant direct methods
Least-squares matrix: full	Secondary atom site location: difference Fourier map
*R*[*F*^2^ > 2σ(*F*^2^)] = 0.057	Hydrogen site location: inferred from neighbouring sites
*wR*(*F*^2^) = 0.152	H-atom parameters constrained
*S* = 1.00	*w* = 1/[σ^2^(*F*_o_^2^) + (0.0622*P*)^2^] where *P* = (*F*_o_^2^ + 2*F*_c_^2^)/3
2313 reflections	(Δ/σ)_max_ < 0.001
191 parameters	Δρ_max_ = 0.19 e Å^−^^3^
0 restraints	Δρ_min_ = −0.22 e Å^−^^3^

### Special details

**Table:**

Geometry. Bond distances, angles *etc*. have been calculated using the rounded fractional coordinates. All su's are estimated from the variances of the (full) variance-covariance matrix. The cell e.s.d.'s are taken into account in the estimation of distances, angles and torsion angles
Refinement. Refinement of *F*^2^ against ALL reflections. The weighted *R*-factor *wR* and goodness of fit *S* are based on *F*^2^, conventional *R*-factors *R* are based on *F*, with *F* set to zero for negative *F*^2^. The threshold expression of *F*^2^ > σ(*F*^2^) is used only for calculating *R*-factors(gt) *etc*. and is not relevant to the choice of reflections for refinement. *R*-factors based on *F*^2^ are statistically about twice as large as those based on *F*, and *R*- factors based on ALL data will be even larger.

### Fractional atomic coordinates and isotropic or equivalent isotropic displacement parameters (Å^2^)

**Table:**

	*x*	*y*	*z*	*U*_iso_*/*U*_eq_	
O1	1.2830 (3)	0.00635 (16)	0.14061 (19)	0.0527 (10)	
O2	1.3616 (3)	0.01396 (16)	0.34045 (19)	0.0529 (10)	
O3	1.1429 (3)	0.07152 (17)	0.44973 (17)	0.0486 (9)	
N1	0.6066 (4)	0.10087 (18)	0.0169 (2)	0.0394 (10)	
N2	0.1653 (4)	0.11644 (18)	−0.3115 (2)	0.0408 (10)	
C1	1.2446 (5)	0.0237 (2)	0.2380 (3)	0.0375 (12)	
C2	1.0566 (4)	0.0584 (2)	0.2369 (3)	0.0334 (11)	
C3	1.0142 (5)	0.0819 (2)	0.3454 (3)	0.0364 (11)	
C4	0.8368 (5)	0.1138 (2)	0.3417 (3)	0.0459 (13)	
C5	0.7012 (5)	0.1219 (2)	0.2364 (3)	0.0424 (14)	
C6	0.7423 (4)	0.0983 (2)	0.1288 (3)	0.0338 (11)	
C7	0.9190 (4)	0.0666 (2)	0.1312 (3)	0.0349 (11)	
C8	0.4406 (5)	0.1355 (2)	−0.0070 (3)	0.0387 (11)	
C9	0.3080 (5)	0.1370 (2)	−0.1184 (3)	0.0344 (11)	
C10	0.1205 (4)	0.1715 (2)	−0.1405 (3)	0.0324 (11)	
C11	0.0164 (5)	0.2123 (2)	−0.0705 (3)	0.0410 (11)	
C12	−0.1644 (5)	0.2377 (2)	−0.1229 (3)	0.0451 (12)	
C13	−0.2477 (5)	0.2216 (2)	−0.2434 (3)	0.0476 (14)	
C14	−0.1494 (5)	0.1805 (2)	−0.3140 (3)	0.0411 (11)	
C15	0.0346 (5)	0.1569 (2)	−0.2617 (3)	0.0350 (11)	
C16	0.3256 (5)	0.1038 (2)	−0.2278 (3)	0.0411 (12)	
H1	0.63809	0.07659	−0.04221	0.0472*	
H2	0.14581	0.10162	−0.38548	0.0489*	
H3	1.24072	0.05315	0.43705	0.0584*	
H4	0.80901	0.13014	0.41293	0.0551*	
H5	0.58271	0.14295	0.23633	0.0512*	
H7	0.94585	0.05043	0.05962	0.0420*	
H8	0.40583	0.16216	0.05637	0.0463*	
H11	0.06906	0.22218	0.01060	0.0494*	
H12	−0.23290	0.26623	−0.07704	0.0542*	
H13	−0.37122	0.23872	−0.27642	0.0567*	
H14	−0.20470	0.16904	−0.39435	0.0495*	
H16	0.43238	0.07717	−0.24027	0.0491*	

### Atomic displacement parameters (Å^2^)

**Table:**

	*U*^11^	*U*^22^	*U*^33^	*U*^12^	*U*^13^	*U*^23^
O1	0.0458 (16)	0.076 (2)	0.0390 (14)	0.0109 (13)	0.0160 (12)	−0.0088 (13)
O2	0.0373 (15)	0.079 (2)	0.0397 (15)	0.0091 (13)	0.0048 (12)	−0.0035 (14)
O3	0.0402 (16)	0.0743 (19)	0.0303 (13)	0.0077 (14)	0.0070 (11)	−0.0027 (12)
N1	0.0406 (19)	0.0466 (19)	0.0307 (15)	0.0039 (16)	0.0087 (13)	−0.0042 (14)
N2	0.0439 (19)	0.0481 (19)	0.0292 (15)	0.0048 (15)	0.0071 (14)	−0.0045 (14)
C1	0.036 (2)	0.040 (2)	0.035 (2)	0.0021 (17)	0.0063 (17)	−0.0006 (17)
C2	0.034 (2)	0.036 (2)	0.0306 (18)	−0.0016 (16)	0.0087 (15)	−0.0013 (16)
C3	0.037 (2)	0.041 (2)	0.0285 (18)	0.0015 (18)	0.0033 (16)	0.0003 (16)
C4	0.050 (2)	0.060 (3)	0.0279 (18)	0.008 (2)	0.0104 (17)	−0.0090 (18)
C5	0.037 (2)	0.054 (3)	0.036 (2)	0.0112 (18)	0.0086 (16)	−0.0048 (18)
C6	0.035 (2)	0.038 (2)	0.0260 (17)	0.0012 (17)	0.0031 (15)	−0.0027 (16)
C7	0.034 (2)	0.042 (2)	0.0295 (18)	0.0013 (16)	0.0093 (15)	−0.0038 (16)
C8	0.041 (2)	0.040 (2)	0.0348 (19)	0.0065 (18)	0.0090 (16)	0.0011 (17)
C9	0.036 (2)	0.037 (2)	0.0288 (17)	0.0008 (16)	0.0056 (15)	−0.0030 (16)
C10	0.037 (2)	0.031 (2)	0.0279 (18)	−0.0014 (16)	0.0058 (15)	0.0007 (15)
C11	0.048 (2)	0.042 (2)	0.0332 (19)	0.0006 (19)	0.0109 (17)	−0.0011 (17)
C12	0.044 (2)	0.048 (2)	0.047 (2)	0.0103 (19)	0.0181 (18)	0.0001 (19)
C13	0.040 (2)	0.056 (3)	0.045 (2)	0.0095 (19)	0.0075 (18)	0.004 (2)
C14	0.043 (2)	0.047 (2)	0.0308 (19)	0.0011 (19)	0.0047 (17)	0.0003 (17)
C15	0.037 (2)	0.034 (2)	0.0352 (19)	0.0039 (17)	0.0115 (16)	0.0028 (16)
C16	0.037 (2)	0.046 (2)	0.038 (2)	0.0066 (18)	0.0050 (17)	−0.0019 (18)

### Geometric parameters (Å, °)

**Table:**

O1—C1	1.254 (4)	C9—C10	1.440 (5)
O2—C1	1.283 (4)	C9—C16	1.399 (5)
O3—C3	1.339 (4)	C10—C15	1.399 (5)
O3—H3	0.8200	C10—C11	1.397 (5)
N1—C6	1.418 (4)	C11—C12	1.371 (5)
N1—C8	1.298 (5)	C12—C13	1.392 (5)
N2—C16	1.335 (4)	C13—C14	1.375 (5)
N2—C15	1.388 (5)	C14—C15	1.384 (5)
N1—H1	0.8600	C4—H4	0.9300
N2—H2	0.8600	C5—H5	0.9300
C1—C2	1.480 (5)	C7—H7	0.9300
C2—C7	1.379 (5)	C8—H8	0.9300
C2—C3	1.412 (5)	C11—H11	0.9300
C3—C4	1.385 (5)	C12—H12	0.9300
C4—C5	1.368 (5)	C13—H13	0.9300
C5—C6	1.398 (5)	C14—H14	0.9300
C6—C7	1.382 (4)	C16—H16	0.9300
C8—C9	1.403 (5)		
			
C3—O3—H3	109.00	C9—C10—C11	134.9 (3)
C6—N1—C8	127.8 (3)	C10—C11—C12	119.1 (3)
C15—N2—C16	110.1 (3)	C11—C12—C13	121.4 (3)
C8—N1—H1	116.00	C12—C13—C14	120.7 (3)
C6—N1—H1	116.00	C13—C14—C15	117.8 (3)
C16—N2—H2	125.00	C10—C15—C14	122.5 (3)
C15—N2—H2	125.00	N2—C15—C10	107.6 (3)
O2—C1—C2	117.3 (3)	N2—C15—C14	129.9 (3)
O1—C1—O2	123.3 (3)	N2—C16—C9	109.4 (3)
O1—C1—C2	119.4 (3)	C3—C4—H4	119.00
C3—C2—C7	118.9 (3)	C5—C4—H4	119.00
C1—C2—C3	120.1 (3)	C4—C5—H5	120.00
C1—C2—C7	121.0 (3)	C6—C5—H5	120.00
O3—C3—C4	121.1 (3)	C2—C7—H7	119.00
O3—C3—C2	120.1 (3)	C6—C7—H7	119.00
C2—C3—C4	118.8 (3)	N1—C8—H8	117.00
C3—C4—C5	121.9 (3)	C9—C8—H8	117.00
C4—C5—C6	119.4 (3)	C10—C11—H11	120.00
C5—C6—C7	119.2 (3)	C12—C11—H11	120.00
N1—C6—C5	122.7 (3)	C11—C12—H12	119.00
N1—C6—C7	118.0 (3)	C13—C12—H12	119.00
C2—C7—C6	121.7 (3)	C12—C13—H13	120.00
N1—C8—C9	126.8 (3)	C14—C13—H13	120.00
C8—C9—C10	125.5 (3)	C13—C14—H14	121.00
C8—C9—C16	128.2 (3)	C15—C14—H14	121.00
C10—C9—C16	106.3 (3)	N2—C16—H16	125.00
C11—C10—C15	118.4 (3)	C9—C16—H16	125.00
C9—C10—C15	106.7 (3)		

### Hydrogen-bond geometry (Å, °)

**Table:**

Cg3 is the centroid of the C10–C15 ring.

**Table:**

*D*—H···*A*	*D*—H	H···*A*	*D*···*A*	*D*—H···*A*
N1—H1···O1^i^	0.86	1.90	2.735 (3)	162
N2—H2···O3^ii^	0.86	1.95	2.805 (3)	171
O3—H3···O2	0.82	1.70	2.448 (3)	150
C16—H16···O1^i^	0.93	2.48	3.279 (4)	144
C16—H16···O2^i^	0.93	2.55	3.439 (4)	160
C5—H5···Cg3^iii^	0.93	2.92	3.643 (4)	136

Symmetry codes: (i) −*x*+2, −*y*, −*z*; (ii) *x*−1, *y*, *z*−1; (iii) *x*+1/2, −*y*+1/2, *z*+1/2.
